# FAST-FOOD RESTAURANTS: A NEIGHBORHOOD RESOURCE FOR COGNITIVE FUNCTION AMONG AGING AMERICANS?

**DOI:** 10.1093/geroni/igz038.103

**Published:** 2019-11-08

**Authors:** Jessica M Finlay, Philippa Clarke, Mike Esposito, Iris Gomez-Lopez, Suzanne Judd, Virginia Wadley

**Affiliations:** 1 Social Environment and Health, Institute for Social Research, University of Michigan, Ann Arbor, Michigan, United States; 2 Institute for Social Research, University of Michigan, Ann Arbor, Michigan, United States; 3 The University of Alabama at Birmingham, Birmingham, Alabama, United States

## Abstract

In this exploratory mixed-methods sequential design study, interviews with 125 adults aged 55-92 (mean age 71) living in the Minneapolis (Minnesota) metropolitan area suggest that large-chain fast-food restaurants such as McDonald’s may serve as reservoirs of cognitive function. Thematic analysis revealed perceived benefits of fast-food settings for older adults including familiarity and comfort; affordability; sociability with friends, family, staff, and customers; and entertainment (e.g., newspapers, crosswords). To further test these observations, we analyzed data from urban and suburban REGARDS participants. Preliminary multilevel regression models found that participants residing within 5 kilometers of a McDonald’s restaurant exhibited higher cognitive function than similar individuals who live further from said organizations (b=0.31; se=0.12). The results complicate understanding of fast-food settings and prompt further research that tests whether restaurants can serve as community spaces for older adults to help buffer against cognitive decline by fostering social interaction and mental stimulation.

